# Oncogenomic Approaches in Exploring Gain of Function of Mutant p53

**DOI:** 10.2174/138920208784340713

**Published:** 2008-05

**Authors:** Sara Donzelli, Francesca Biagioni, Francesca Fausti, Sabrina Strano, Giulia Fontemaggi, Giovanni Blandino

**Affiliations:** 1 Department of Experimental Oncology, Regina Elena Cancer Institute, 00158-Rome, Italy; 2 Rome Oncogenomic Center, 00158-Rome, Italy

## Abstract

Cancer is caused by the spatial and temporal accumulation of alterations in the genome of a given cell. This leads to the deregulation of key signalling pathways that play a pivotal role in the control of cell proliferation and cell fate. The p53 tumor suppressor gene is the most frequent target in genetic alterations in human cancers. The primary selective advantage of such mutations is the elimination of cellular wild type p53 activity. In addition, many evidences *in vitro* and *in vivo* have demonstrated that at least certain mutant forms of p53 may possess a gain of function, whereby they contribute positively to cancer progression. The fine mapping and deciphering of specific cancer phenotypes is taking advantage of molecular-profiling studies based on genome-wide approaches. Currently, high-throughput methods such as array-based comparative genomic hybridization (CGH array), single nucleotide polymorphism array (SNP array), expression arrays and ChIP-on-chip arrays are available to study mutant p53-associated alterations in human cancers. Here we will mainly focus on the integration of the results raised through oncogenomic platforms that aim to shed light on the molecular mechanisms underlying mutant p53 gain of function activities and to provide useful information on the molecular stratification of tumor patients.

## INTRODUCTION

**Fig. (1):**
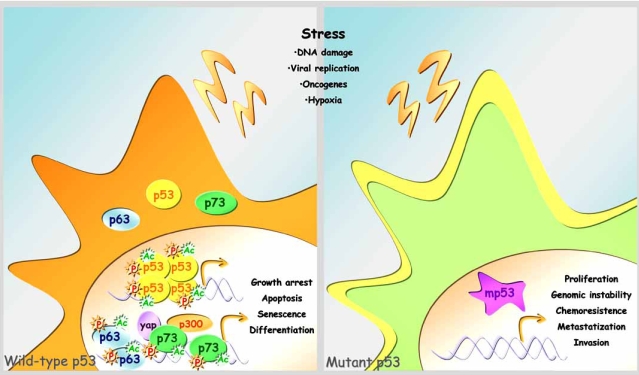
**Wild type versus mutant p53 protein.** In response to various types of cellular stress signals, wild-type p53 protein and its family members, p63 and p73, undergo different kinds of post-translational modifications, including phosphorylation and acetylation. These events cause p53 family members to stabilize and activate which results in growth arrest, apoptosis, senescence, and differentiation. However, cells carrying mutant p53 display increased cellular proliferation, genomic instability, chemoresistance, metastatization and invasion.

The p53 tumor suppressor protein is a sequence-specific transcription factor that is present in a latent form under normal conditions and becomes activated by a variety of stress signals, such as DNA damage, oncogene activation and improper mitogenic stimulation. p53 acts as a potent transcriptional activator through the binding as a homotetramer to specific sequences, named p53 responsive elements (p53RE). Activated p53 modulates the expression of a large set of target genes involved in many cellular processes, including cell cycle arrest, DNA repair, apoptosis and senescence. Growth arrest or cell death prevents damaged DNA from being replicated; thus suggesting a role for p53 in the maintenance of genome integrity [1-5]. p53 is a member of a family of proteins that has recently been established through the identification of p73, p63 and their related isoforms, all heavily involved in tumor suppression and development [6]. Half of all human cancers bears p53 mutations [7]. The most prevalent type of p53 alterations are missense mutations, often within the conserved DNA binding core domain of the protein [8,9]. In this area each residue has been found to be possibly mutated in human tumors, and the resulting proteins display a marked heterogeneity in terms of loss of structure and function. Several evidences demonstrate that a subset of p53 mutant proteins display gain of function activity [10], thereby actively participating to tumorigenesis (Fig. **1**). Structural studies have allowed the classification of p53 mutant proteins in two major categories [11,12]: a) DNA contact defective (His273, Trp248), that includes those mutants whose mutation impacts on the residues composing the DNA/protein interaction surface; b) defective structure (His175, His179), whose point mutation determines an important conformational alteration. At variance with the observations of several other tumor suppressor genes, cells with p53 mutations typically maintain expression of full-length mutant protein, often at markedly elevated levels. This observation suggests the possibility that some mutant p53 proteins gain additional functions over the mere loss of wild-type TP53 activity. This hypothesis has been validated by a combination of *in vitro* and *in vivo* studies that show how mutant p53 proteins confer transformed properties to cell cultures and increased tumorigenicity in mice [13,14]. It has also been reported that conformational mutants, such as p53His175, but not DNA contact mutants (p53His273, p53Trp248), can increase cellular resistance to etoposide or can contribute to genomic instability by abrogating the mitotic spindle checkpoint, thereby facilitating the generation of aneuploid cells [15,16]. p53-/- mice develop a different spectrum of tumours compared to that of knock-in mice expressing various p53 hot spot mutants [15,17]. Indeed, mutant p53 knock-in mice have a higher frequency of solid tumours with high potential for metastasis, a feature not seen in knock-out animals. This peculiar spectrum of tumours is also observed in mice expressing one mutant p53 allele in a p53-null background, thus strongly suggesting a gain of function of mutant p53 proteins. Further analysis of mutant p53 knock-in models in mice defective in p63 and p73 suggests that alteration in the activity of the entire p53 family is an important feature in achieving function of mutant p53. Many questions regarding the molecular mechanisms at the basis of gain of function activities are still unsolved.

**Fig. (2). Molecular mechanisms underlying gain of function of mutant p53 protein.**
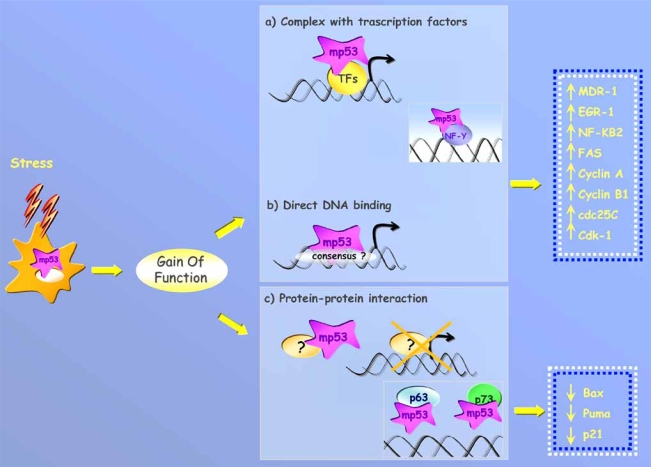
**DNA binding.** Mutant p53 protein can be recruited on the promoters of its target genes by the interaction with different transcription factors, as has been demonstrated for the transcription factor NF-Y in response to DNA damage (a), or by direct binding to specific consensus sequences that are still to be identified (b). **Protein-protein interaction.** (c) Mutant p53 protein binds to and sequesters proteins (p73 and p63) whose activities are closely connected to anti-tumoral effects such as apoptosis or growth inhibition.

To date, we can depict the three following molecular scenarios to explain gain of function of mutant p53 proteins. **(a)** mutant p53 can bind to DNA through the association with DNA binding proteins and transcriptionally activate specific target genes using its functional transactivation domain (TAD). In support of this molecular mechanism, it has been reported that human tumor-derived p53 mutants, whose TAD was inactivated by site-directed mutagenesis, lost the ability to increase tumorigenicity *in vitro* and *in vivo* [8,13, 19-21]; **(b)** recent work by Di Agostino *et al*. has shown that mutant p53 proteins physically interact *in vivo *with the transcription factor NF-Y, whose DNA binding consensus are present in the regulatory regions of many key genes involved in the regulation of the cell cycle. Mutant p53 can be recruited *in vivo *onto the promoter of NF-Y target genes, as a member of a large transcriptional complex that also includes histone acetyltransferases (HAT). Of note, mutant p53 favours the selective recruitment of HAT in response to DNA damage, thereby dictating the transcriptional activation of its target genes [22].** (c)** mutant p53 binds to and sequesters proteins whose function is required for anti-tumor effects such as apoptosis or growth inhibition. Interestingly, it has been reported that human tumor-derived p53 mutants can associate with p73 and p63 and interfere with their transcriptional activity and ability to induce apoptosis when co-expressed in transient transfection assays (Fig. **2**) [19,23-26].

Many of the studies exploring the molecular mechanisms that underlie gain of function of mutant p53 proteins have mainly characterized the activities of p53 hot spot mutations. These mutants display an oncogenic potential by themselves, as demonstrated by transformation assays *in vitro* and knock-in models *in vivo*. However, as mentioned above, mutations can occur within the entire DBD of the protein. The activity of a large number of p53 mutants has been analyzed by Kato *et al*. [27]. This analysis demonstrated that hot spot mutations lost their transactivation activity completely, while the rest of the mutants retained some ability to transactivate target genes. In parallel to these observations, additional evidences have shown that the clinical penetrance of p53 mutations in cancer is strongly influenced by the genetic background of the individual [13,17,28] and by the presence of other cancer-associated somatic mutations [29].

Since p53 mutations of the DBD are selected in cancer, it is reasonable to hypothesize that hot spot mutations are directly selected because they represent the driving force during the neoplastic transformation. The remaining p53 mutations are selected following a second mutational event that might be permissive for mutant p53 gain of function activities. Cell-type specificity for gain of function of mutant p53 might explain why certain p53 mutants do not display any activity when exogenously overexpressed in cell systems different from those where the mutation was originated. 

## p53 MUTATIONS AS PREDICTIVE/PROGNOSTIC MARKERS IN HUMAN CANCERS

Since the first description of a p53 mutation in human cancers in 1989, several thousands of papers have described clinical studies in which p53 has been tentatively linked to the response to treatment (predictive power) or patient survival (prognostic power). The usefulness of these studies was questioned in terms of strategy (too few patients enrolled for correct statistical analysis) and methodology [30,31]. Indeed, the majority of the studies have relied on immunohistochemistry to assess p53 alterations. This approach is a poor surrogate for gene mutation detection since many mutations do not lead to protein accumulation, and also because an accumulation of wild type p53 might occur. Langerod *et al*. [32] have shown how IHC detects 50% of the p53 mutations identified through TTGE (temporal temperature gradient gel electrophoresis). With the introduction of gene sequencing to precisely identify p53 mutations in cancer, it has been clearly defined that the presence of a p53 mutation is correlated with a shorter survival or a poor response to treatment in several cancers. Moreover, mutations within the DNA binding domain have been associated with a worst prognosis compared to that related to other p53 mutations in breast cancer [33-36]. Now that the power of mutant p53 as a predictive/prognostic marker in breast cancer has been established, the complete characterization of mutant p53 contribution to tumorigenesis will require a combination of oncogenomic approaches, for example copy number analyses, mutational studies and expression profiling (protein coding genes and microRNAs) on a large number of patients.

## MUTANT p53 AND CHROMOSOMAL INSTABILITY

The genome of cancer cells is thought to be unstable as they often harbour many chromosomal abnormalities. Due to the role of TP53 in maintaining genomic stability, its inactivation profoundly impairs repair of DNA damage and could promote genomic alterations. Several research groups have reported a correlation between genomic instability and TP53 mutations in breast cancer tissues. Using fluorescence *in situ* hybridization, Sigurdsson *et al*. [37] have shown that breast cancer cells with abnormal p53 display a higher copy number of chromosome 17 than cells without p53 staining. This points to a relationship between TP53 alterations and chromosomal instability. Jain *et al*. [38] combined information on TP53 mutation status in breast carcinomas with their genomic complexity, as assessed by chromosomal comparative genomic hybridization (CGH), a technique capable of detecting copy number alterations at chromosomal level [39]. They found that gain at 8q24 and loss at 5q15-5q21 are linked to mutant p53. Subsequently, Jong *et al*. [40] and Kleivi *et al*. [41] performed CGH analysis in relation to TP53 status on breast cancer specimens and confirmed a higher degree of abnormalities in patients carrying TP53 mutation, compared to those with wild type p53. These abnormalities appeared to cluster in certain chromosomal regions at significantly higher frequencies. Of note, the result of 8q gain associated with the TP53 mutated status was shared by all three studies. Identification of putative cancer-related genes that reside within these genomic loci, such as c-myc (at 8q24), and their potential interactions with mutant p53 might represent an exciting new area for breast cancer research.

**Fig. (3):**
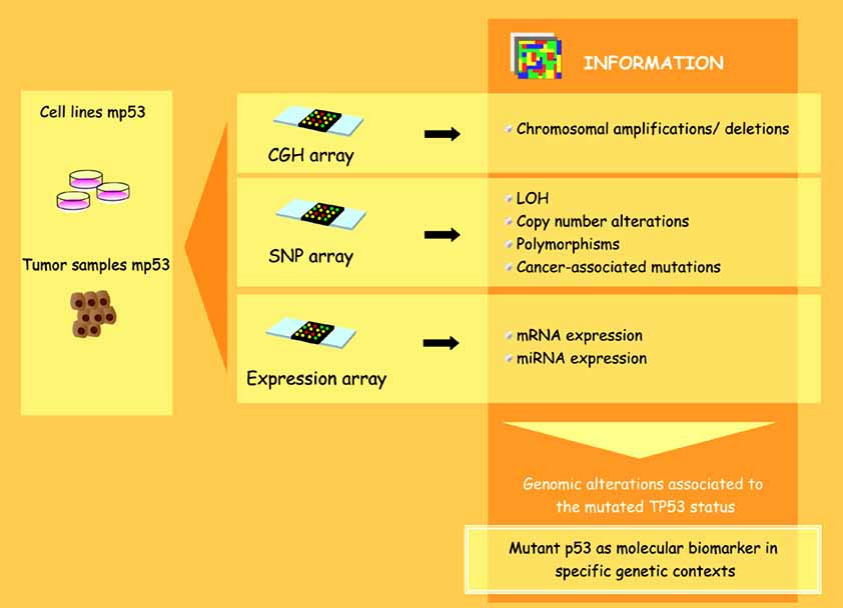
**Oncogenomic approaches.** Different kinds of oncogenomic approaches can be used to analyze genomic alterations in cell lines or tumor samples carrying mutant p53 proteins: *CGH arrays*, which allow the identification of chromosomal amplifications and deletions, and *SNP arrays*, by which it is possible to analyze the presence of LOH, copy number alterations, polymorphisms and cancer-associated mutations.

Currently, high throughput methods such as array-based comparative genomic hybridization (CGH array) and single nucleotide polymorphism array (SNP array) are available to study mutant p53-associated alterations in a genome-wide fashion. Array-CGH is an established high-resolution method to study the whole genome for chromosomal amplifications and deletions [42]. One limitation of the array-CGH method is its lack of genotype information. Subsequently, it fails to provide information about regions of LOH without copy number alteration (CNA) such as mitotic recombination or gene conversion events. Recently, high-density oligonucleotide-based single polymorphism arrays (SNP arrays) have been used to identify copy number and LOH of chromosomal regions. The advantage of a combined SNP-CGH approach is the identification of allele specific gain/loss by SNP array and the robust copy number detection by array CGH. In addition to the copy number detection, SNP array would also lead to an increase in information about the genetic background of the analysed patients and about additional cancer-associated mutations. For example, this method would reveal the presence of specific SNP variants in components of p53-interacting pathways. The integration of these two platforms (CGH, SNP) with expression arrays will provide detailed and relevant information about the genomic alterations associated with the mutated TP53 status and about the consequences determined by these alterations at the expression level. It is still being debated as to whether copy number alterations lead to changes in gene expression. So far, few studies have indicated that a correlation exists between chromosomal instability and gene expression in colorectal carcinoma and other cancer types [43,44]. Moreover, if particular associations between specific p53 mutations and other genomic alterations will be shown to be related to the clinical outcome of given tumors, it is highly possible that these combined analyses would reinforce the value of mutant p53 as a molecular biomarker in specific genetic contexts (Fig. **3**). 

## IDENTIFICATION OF mtp53-ASSOCIATED GENE-EXPRESSION SIGNATURES

Two different approaches have been followed to identify mutant p53-associated gene-expression signatures: **a**) cell lines overexpressing different mutant forms of p53 have been generated to analyze the impact of mutant p53 on cell transcriptome; **b**) tumor samples with characterized p53 status (mutated or wild-type) have been examined by microarray analysis for their global gene expression. The latter has been compared with that of normal counterparts to define (mainly through hierarchical clustering or uninvariate analysis by SAM) clusters of genes associated with the mutated status of p53.

A number of studies employed microarrays to evaluate global gene expression in H1299 lung cancer cells expressing p53-175H, p53-273H and p53-281G [45-47], p53-deficient HCT116 colorectal cancer cells overexpressing p53-138P and p53-175H [48], osteosarcoma U2OS cells expressing p53-157F, p53-175H and p53-248Q [49], LNCaP prostate cancer cells expressing p53-245S, p53-248W, p53-273C, p53-273H [50] as well as in Li Fraumeni syndrome-derived fibroblasts expressing p53-175H [51] have been performed. The main limitation of these studies is based on the fact that, except for the O’Farrell study [48], they failed to analyze the impact of wt p53 expression on the same cell system. For this reason, they were unable to assess whether some of the transcriptional modifications observed would have been found, to a different or opposite extent, upon expression of wild-type p53. Indeed, O’Farrell *et al*. report that the most obvious difference related to the ectopic expression of two diverse p53 mutants (R175H and A138P) is that R175H retained less wild-type transcriptional regulatory events than A138P. A long list of mutant p53-regulated genes has been obtained from microarray analyses, suggesting the involvement of mutant p53 in several cellular processes ranging from transcriptional and translational regulation, signal transduction, cell motility, invasion and metastatization. This list obviously includes some of the genes previously characterized as mutant p53 targets. Importantly, all the identified genes provide evidence for the potential disclosure of new molecular targets and for substantial insights into the molecular mechanisms underlying gain of function activities of mutant p53.

In addition to the *in vitro* models of cell lines overexpressing p53 mutants, the impact of a mutated p53 on the transcriptome has also been evaluated through *in vivo* microarray analyses. As mentioned above, the mutational status of p53 is prognostic in many malignancies. In breast cancer, p53 mutations are associated with worse overall and disease-free survival rates and have been implicated in resistance to anticancer therapies. Miller *et al*. [52] reported on a microarray analysis performed on 251 primary invasive breast tumors with known p53 status (58 carrying mutant p53 and 193 wild-type). They found that tumors with mutated and wild-type p53 are distinguished by pervasive molecular differences (mainly in genes controlling proliferation). They also identified a 32-gene expression signature (as optimal classifier) that distinguishes mutant versus wild-type p53 tumors of different histological types. Furthermore, it also enables the prediction of the p53 status when tested on two publicly available microarray data sets (breast cancer [53], and liver cancer [54]) where p53 mutational status is known. The identified p53 signature predicts outcome better than p53 mutation status alone, also independently from the therapy-specific data sets.

Despite the efficacy of these *in vivo* analyses in identifying gene expression signatures associated with wild type or mutant p53, unfortunately these studies are unable to assess whether the characterized genes are really dependent on the presence of mutant p53 or whether they merely represent the result of a loss of wild-type p53 activity. The data sets derived from microarray analyses on cell lines overexpressing p53 mutants, enclosing the putative genes responsible for gain of function, need to be matched with those derived from *in vivo* analyses to define which of the transcripts are truly expressed in cancer tissues carrying p53 mutation. Up until now few p53 mutations have been analyzed *in vitro* for their ability to transcriptionally affect gene expression. Further microarray analyses employing additional p53 mutants to be compared with those performed on tumor specimens with known p53 status might unveil important molecular details on the gain of function properties of the different mutant p53 proteins present in human cancers.

## ChIP-CHIP AS A TOOL TO IDENTIFY mut-p53 TRANSCRIPTIONAL SIGNATURES IN HUMAN CANCERS

Little is known about the mechanisms through which mutant p53 proteins, that have lost the ability to bind DNA in a sequence-specific manner, can achieve the specificity in gene regulation. Chromatin Immunoprecipitation experiments have shown that mutant p53 can be found onto the regulatory regions of putative target genes, but despite extensive efforts, the identification of the specific DNA binding consensus of mutant p53 has not yet been found. It has been proposed that mutant p53 can be recruited to its putative target promoters through the physical interaction with sequence specific transcription factors whose specific DNA binding consensus are present on those regulatory regions. Sp1 and Ets, which were shown to cooperate with mutant p53 in transcriptional regulation [55-57], are paradigmatic of the proposed molecular mechanism. The notion that mutant p53 is recruited to transcriptional regulatory regions of peculiar genes *via* interaction with other transcription factors is strongly supported by the work of Di Agostino *et al*. [22] which has shown the existence of a transcriptional competent protein complex involving mutant p53 and the transcription factor NF-Y. Along this transcriptional pathway mutant p53 favours the recruitment of the coactivator p300 onto NF-Y target promoters, leading to the transcriptional activation of its target genes. Our group has recently also found that the transcription factors E2F-1 and p65 (RelA) are both concomitantly present with mutant p53 on the promoters of some putative target genes (G.F. and G.B. unpublished observations).

Due to the absence of a canonical consensus sequence for mutant p53, the only approach that seems truly promising for the precise deciphering of its transcriptional code is the use of whole-genome tiling arrays, designed to interrogate an entire genome in an unbiased fashion, for ChIP-chip analysis. ChIP-chip (Chromatin Immunoprecipitation-chip) permits a simultaneous analysis of the occupancy of a specific transcription factor on thousands of its target genes. Briefly, crosslinked chromatin (DNA/protein) complexes are extracted from the analyzed cell culture or from a tissue and sheared down to relatively short fragments (500-1000 bp). The chromatin fragments containing the transcription factor of interest are then immunoprecipitated using a specific antibody and the target sequences are subsequently identified through hybridization to DNA microarrays. At a high probe tiling resolution multiple overlapping probes may contain the actual transcription factor binding motif and thus enable a fine mapping of the binding site to a resolution of less than 25 bp. Apart from the most recent generated tiling arrays, different kinds of microarrays have been generated for ChIP-chip analysis. Initial studies were performed using slides carrying spotted oligonucleotides or PCR products from selected predicted promoters. However these arrays are unable to interrogate the entire genome and are unable to address the possibility that transcription factors might bind to other locations. Recent studies using tiling arrays interrogating entire chromosomes show that a large fraction of *in vivo* binding sites are outside the predicted promoter regions of given genes. It has been shown that the binding locations of NFkB, cMyc, Sp1, p53, CREB are found within both coding and non-coding regions. More binding sites than those predicted were found and of those only a relatively small fraction of the sites occurred in genomic regions that would typically be considered “promoters”. The use of tiling arrays in the study of mutant p53 transcriptional activity will overcome the difficulties existing in the prediction of the binding sites for this oncogenic transcription factor. In addition, mutant p53 has been reported to bind a wide range of DNA secondary structures. For example, mutant p53 was shown to preferentially bind matrix attachment regions (MARs) [58]. MARs are involved in the anchoring of chromatin fibres to the nuclear matrix, generating chromatin domains that may enhance or repress transcription. Further studies from this group suggest that the binding to non-B DNA rather than a specific sequence represents the basis of the interaction between mutant p53 and MARs. If the binding of mutant p53 to MARs leads to changes in chromatin accessibility and activity it becomes more necessary to identify the genes that will sense the effects of this non-sequence-specific binding. This information will likely be available after a hybridization of whole-genome tiling arrays with mutant p53-bound chromatin.

**Fig. (4):**
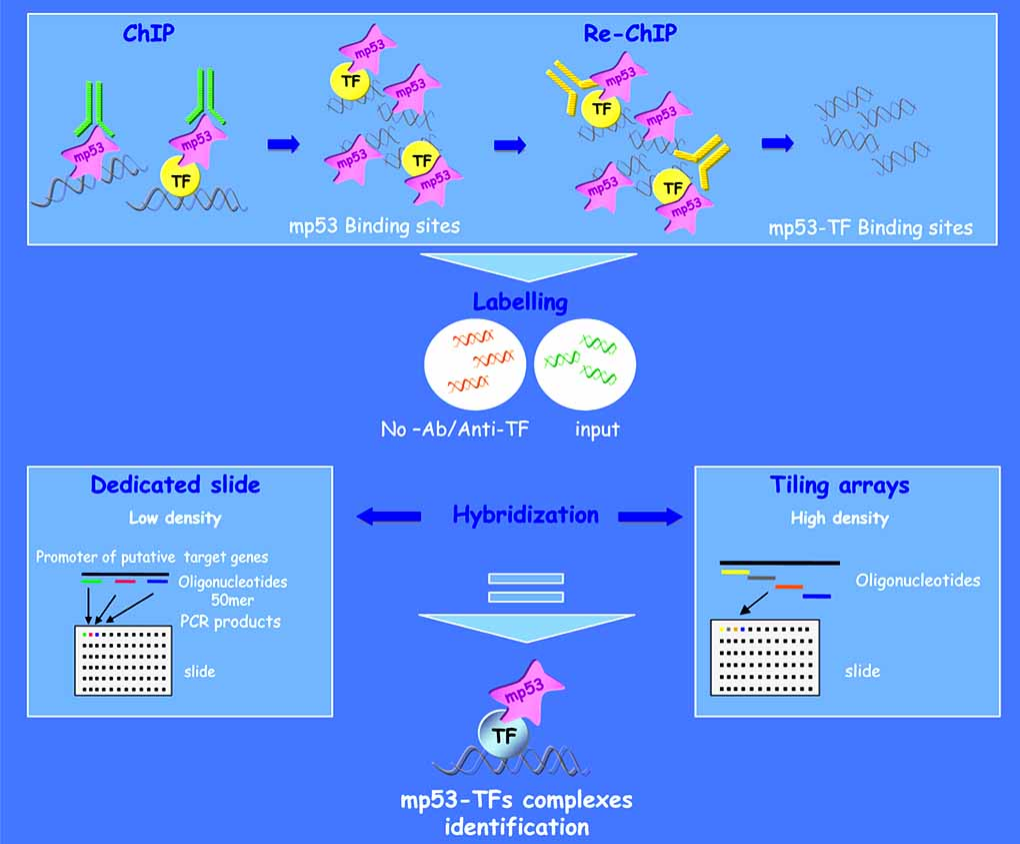
**ChIP-chip approach.** ChIP-chip technique allows for the simultaneous analysis of the presence of a specific transcription factor on the regulatory regions of thousands of its putative target genes. ChIP-chip analysis of chromatin immunoprecipitated with an antibody against p53 and subsequently re-immunoprecipitated with an antibody against a specific transcription factor (Re-ChIP), in a mutant p53 cellular context, allows for the identification of a transcriptional protein complex that might control the activation or repression of a target gene. Two different types of slides can be used for the ChIP-chip analysis: *dedicated slides* containing oligonucleotide probes specific for different regions of known and putative target gene promoters; *tiling slides* containing oligonucleotide probes representing the entire genomic sequences.

The ChIP-chip analysis of chromatin initially immunoprecipitated with antibodies against mutant p53 and successively with antibodies directed to the various transcription factors that mediate mutant p53’s binding to its target genes (Re-ChIP) is likely to lead to the identification of subgroups of target genes controlled by different mutant p53-TF protein complexes (Fig. **4**).

## CONCLUSIONS

Genome wide analyses are turning out to be powerful instruments in the molecular stratification of cancer patients. The complexity of the data raised through the oncogenomic approaches calls for the integration of multidisciplinary expertises and potentially accounts for the fine deciphering and molecular characterization of specific cancer patient phenotypes. An understanding of the molecular events regulating and/or governed by gain of function mutant p53 proteins might lead to the disclosure of cancer signalling pathways to be tackled with novel anticancer therapies.

## References

[1] Harris SL, Levine AJ (2005). The p53 pathway: positive and negative feedback loops. Oncogene.

[2] Oren M (2003). Decision making by p53: life, death and cancer. Cell Death Differ.

[3] Toledo F, Wahl GM (2006). Regulating the p53 pathway: *in vitro* hypoteses, *in vivo* veritas. Nat. Rev. Cancer.

[4] Vousden KH, Prives C (2005). P53 and prognosis: new insights and further complexity. Cell.

[5] Beinz B, Zacut-Houri R, Givol D, Oren M (1984). Analysis of the gene coding for the murine cellular tumor antigen p53. EMBO J.

[6] Kaelin Jr WG (1999). The emerging p53 gene family. J. Natl. Cancer Inst.

[7] Kern SE, Kinzler KW, Baker SJ, Nigro JM, Rotter V, Levine AJ, Friedman P, Prives C, Vogelstein B (1989). Mutations in the p53 gene occour in overal human tumor types. Nature.

[8] Hollestein M, Sidransky D, Vogelstein B, Harris CC (1991). P53 mutations in human cancers. Science.

[9] Olivier M, Hussain SP, Cardon DF, Hainaut P, Harris CC (2002). The IARC TP53 database: new online mutation analysis and recommendations to users. Hum. Mutat.

[10] Haley O, Michaloviz D, Oren M (1990). Different tumor-derived p53 mutants exhibit distinct biological activities. Science.

[11] Bullock A, Fersht AR (2001). Rescuing the function of mutant p53. Nat. Rev. Cancer.

[12] Cho YJ, Gorina S, Jeffrey PD, Pavletich NP (1994). Crystal structure of a p53 tumor suppressor DNA complex: understanding tumorigenic mutations. Science.

[13] Olive KP, Tuveson DA, Ruhe ZC, Yin B, Willis NA, Bronson RT, Crowley D, Jacks T (2004). Mutant p53 gain of function in two mouse models of Li-Fraumeni syndrome. Cell.

[14] Jacks T, Remington L, Williams BO, Schmitt EM, Halachmi S, Bronson RT, Weinberg RA (1994). Tumor spectrum analysis in p53-mutant mice. Curr. Biol.

[15] Prives C, Hall PA (1999). The p53 pathway. J. Pathol.

[16] Sigal A, Rotter V (2000). Oncogenic mutations of the p53 tumor suppressor: the demons of the guardian of the genome. Cancer Res.

[17] Lang GA, Iwakuma T, Suh YA, Liu G, Rao VA, Parant JM, Valentin-Vega YA, Terzian T, Caldwell LC, Strong LC, El-Naggar AK, Lozano G (2004). Gain of function of a p53 hot spot mutation in a mouse model of Li-Fraumeni syndrome. Cell.

[18] Blandino G, Levine AJ, Oren M (1999). Mutant p53 gain of function: differential effects of different p53 mutants on resistance of cultured cells to chemotherapy. Oncogene.

[19] Gualberto A, Aldape K, Kozakiewicz K, Tlsty TD (1998). An oncogenic form of p53 confers a dominant, gain-of-function phenotype that disrupts spindle checkpoint control. Proc. Natl. Acad. Sci. USA.

[20] Frazier MW, He X, Wang J, Gu Z, Cleveland JL, Zambetti GP (1998). Activation of c-myc gene expression by tumor-derived p53 mutants requires a discrete c-terminal domain. Mol. Cell. Biol.

[21] Lin J, Teresky AK, Levine AJ (1995). Two critical hydrofobic amino acids in the N-terminal domain of the p53 protein are required for the gain of function phenotypes of human p53 mutants. Oncogene.

[22] Di Agostino S, Strano S, Emiliozzi V, Zerbini V, Mottolese M, Sacchi A, Blandino G, Piaggio G (2006). Gain of function of mutant p53: the mutant p53/NF-Y protein complex reveals an aberrant transcriptional mechanism of cell cycle regulation. Cancer Cell.

[23] Strano S, Munarriz E, Rossi M, Cristofanelli B, Shaul Y, Castagnoli L, Levine AJ, Sacchi A, Cesareni G, Oren M, Blandino G (2000). Physical and functional interaction between p53 mutants and different isoforms of p73. J. Biol. Chem.

[24] Strano S, Fontemaggi G, Costanzo A, Rizzo MG, Monti O, Baccarini A, Del Sal G, Levrero M, Sacchi A, Oren M, Blandino G (2002). Physical interaction with human tumor-derived p53 mutants inhibits p63 activities. J. Biol. Chem.

[25] Strano S, Blandino G (2003). p73-mediated chemosensitivity: a preferential target of oncogenic mutant p53. Cell Cycle.

[26] Di Como CJ, Gaiddon C, Prives C (1999). p73 function is inhibited by tumor-derived p53 mutants in mammalian cells. Mol. Cell. Biol.

[27] Kato S, Han SY, Liu W, Otsuka K, Shibata H, Kanamaru R, Ishioka C (2003). Understanding the function-structure and function-mutation relationships of p53 tumor suppressor protein by high-resolution missense mutation analysis. Proc. Natl. Acad. Sci. USA.

[28] Marin MC, Jost CA, Brooks LA, Irwin MS, O'Nions J, Tidy JA, James N, McGregor JM, Harwood CA, Yulug IG, Vousden KH, Allday MJ, Gusterson B, Ikawa S, Hinds PW, Crook T, Kaelin WG Jr (2000). A common polymorphism act as an intrgenic modifier of mutant p53 behaviour. Nat. Genet.

[29] Soussi T, Wiman KG (2007). Shaping genetic alterations in human cancer: the p53 mutation paradigm. Cancer Cell.

[30] Hall PA, Lane DP (1994). p53 in tumour pathology: can we trust immunohistochemistry?--Revisited!. J Pathol.

[31] Soussi T, Beroud C (2001). Assessing TP53 status in human tumours to evaluate clinical outcome. Nat. Rev. Cancer.

[32] Langerod A, Zhao H, Borgan O, Nesland JM, Bukholm IR, Ikdahl T, Karesen R, Borresen-Dale AL, Jeffrey SS (2007). TP53 mutation status and gene expression profiles are powerful prognostic markers of breast Cancer. Breast Cancer Res.

[33] Borresen AL, Andersen TI, Eyfjord JE, Cornelis RS, Thorlacius S, Borg A, Johansson U, Theillet C, Scherneck S, Hartman S, Cornelisse CJ, Hoving E, Devilee P (1995). TP53 mutations and breast cancer prognosis: particularly poor survival rates for cases with mutations in the zinc-binding domains. Genes Chromosomes Cancer.

[34] Temam S, Flahault A, Perie S, Monceaux G, Coulet F, Callard P, Bernaudin JF, St Guily JL, Fouret P (2000). p53 gene status as a predictor of tumor response to induction chemotherapy of patients with locoregionally advanced squamous cell carcinomas of the head and neck. J. Clin. Oncol.

[35] Olivier M, Langerod A, Carrieri P, Bergh J, Klaar S, Eyfjord J, Theillet C, Rodriguez C, Lidereau R, Bieche I, Varley J, Bignon Y, Uhrhammer N, Winqvist R, Jukkola-Vuorinen A, Niederacher D, Kato S, Ishioka C, Hainaut P, Borresen-Dale AL (2006). The clinical value of somatic TP53 gene mutations in 1,794 patients with breast Cancer. Clin. Cancer Res.

[36] Geisler S, Lnning PE, Aas T, Johnsen H, Haugen DF (2003). TP53 gene mutations predict the response to neoadjuvant treatment with 5-fluorouracil and mitomycin in locally advanced breast Cancer. Clin Cancer Res.

[37] Sigurdsson S, Bodvarsdottir SK, Anamthawat-Jonsson K, Steinarsdottir M, Jonasson JG, Ogmundsdottir HM, Eyfjord JE (2000). p53 abnormality and chromosomal instability in the same breast tumor cells. Cancer Genet. Cytogenet.

[38] Jain AN, Chin K, Borresen-Dale AL, Erikstein BK, Eynstein Lonning P, Kaaresen R, Gray JW (2001). Quantitative analysis of chromosomal CGH in human breast tumors associates copy number abnormalities with p53 status and patient survival. Proc. Natl. Acad. Sci. USA.

[39] Kallioniemi A, Kallioniemi OP, Sudar D, Rutovitz D, Gray JW, Waldman F, Pinkel D (1992). Comparative genomic hybridization for molecular cytogenetic analysis of solid tumors. Science.

[40] Jong YJ, Li LH, Tsou MH, Chen YJ, Cheng SH, Wang-Wuu S, Tsai SF, Chen CM, Huang AT, Hsu MT, Lin CH (2004). Chromosomal comparative genomic hybridization abnormalities in early- and late-onset human breast cancers: correlation with disease progression and TP53 mutations. Cancer Genet. Cytogenet.

[41] Kleivi K, Diep CB, Pandis N, Heim S, Teixeira MR, Lothe RA (2005). TP53 mutations are associated with a particular pattern of genomic imbalances in breast carcinomas. J. Pathol.

[42] Oostlander AE, Meijer GA, Ylstra B (2004). Microarray-based comparative genomic hybridization and its applications in human genetics. Clin. Genet.

[43] Tsafrir D, Bacolod M, Selvanayagam Z, Tsafrir I, Shia J, Zeng Z, Liu H, Krier C, Stengel RF, Barany F, Gerald WL, Paty PB, Domany E, Notterman DA (2006). Relationship of gene expression and chromosomal abnormalities in colorectal Cancer. Cancer Res.

[44] Carter SL, Eklund AC, Kohane IS, Harris LN, Szallasi Z (2006). A signature of chromosomal instability inferred from gene expression profiles predicts clinical outcome in multiple human cancers. Nat. Genet.

[45] Weisz L, Zalcenstein A, Stambolsky P, Cohen Y, Goldfinger N, Oren M, Rotter V (2004). Transactivation of the EGR1 gene contributes to mutant p53 gain of function. Cancer Res.

[46] Scian MJ, Stagliano KE, Deb D, Ellis MA, Carchman EH, Das A, Valerie K, Deb SP, Deb S (2004). Tumor-derived p53 mutants induce oncogenesis by transactivating growth-promoting genes. Oncogene.

[47] Scian MJ, Stagliano KE, Ellis MA, Hassan S, Bowman M, Miles MF, Deb SP, Deb S (2004). Modulation of gene expression by tumor-derived p53 mutants. Cancer Res.

[48] O'Farrell TJ, Ghosh P, Dobashi N, Sasaki CY, Longo DL (2004). Comparison of the effect of mutant and wild-type p53 on global gene expression. Cancer Res.

[49] Mizuarai S, Yamanaka K, Kotani H (2006). Mutant p53 induces the GEF-H1 oncogene, a guanine nucleotide exchange factor-H1 for RhoA, resulting in accelerated cell proliferation in tumor cells. Cancer Res.

[50] Tepper CG, Gregg JP, Shi XB, Vinall RL, Baron CA, Ryan PE, Desprez PY, Kung HJ, deVere White RW (2005). Profiling of gene expression change caused by p53 gain of function mutant alleles in prostate cancer cells. Prostate.

[51] Knaup KX, Roemer K (2004). Cell type-specific regulation of calmodulin 2 expression by mutant p53. FEBS Lett.

[52] Miller LD, Smeds J, George J, Vega VB, Vergara L, Ploner A, Pawitan Y, Hall P, Klaar S, Liu ET, Bergh J (2005). An expression signature for p53 status in human breast cancer predicts mutation status, transcriptional effects, and patient survival. Proc. Natl. Acad. Sci. USA.

[53] Sorlie T, Perou CM, Tibshirani R, Aas T, Geisler S, Johnsen H, Hastie T, Eisen MB, van de Rijn M, Jeffrey SS, Thorsen T, Quist H, Matese JC, Brown PO, Botstein D, Eystein Lonning P, Borresen-Dale AL (2001). Gene expression patterns of breast carcinomas distinguish tumor subclasses with clinical implications. Proc. Natl. Acad. Sci. USA.

[54] Chen X, Cheung ST, So S, Fan ST, Barry C, Higgins J, Lai KM, Ji J, Dudoit S, Ng IO, Van De Rijn M, Botstein D, Brown PO (2002). Gene expression patterns in human liver cancers. Mol. Biol. Cell.

[55] Gualberto A, Baldwin AS Jr (1995). p53 and Sp1 interact and cooperate in the tumor necrosis factor-induced transcriptional activation of the HIV-1 long terminal repeat. J. Biol. Chem.

[56] Chicas A, Molina P, Bargonetti J (2000). Mutant p53 forms a complex with Sp1 on HIV-LTR DNA. Biochem. Biophys. Res. Commun.

[57] Sampath J, Sun D, Kidd VJ, Grenet J, Gandhi A, Shapiro LH, Wang Q, Zambetti GP, Schuetz JD (2001). Mutant p53 cooperates with ETS and selectively up-regulates human MDR1 not MRP1. J. Biol. Chem.

[58] Gohler T, Jager S, Warnecke G, Yasuda H, Kim E, Deppert W (2005). Mutant p53 proteins bind DNA in a DNA structure-selective mode. Nucleic Acids Res.

